# Quality of life and factors affecting it in adult cancer patients undergoing cancer chemotherapy in a tertiary care hospital

**DOI:** 10.1002/cnr2.1312

**Published:** 2020-12-09

**Authors:** Saravana Kumar Ramasubbu, Rajesh K. Pasricha, Uttam K. Nath, Vikram Singh Rawat, Biswadeep Das

**Affiliations:** ^1^ Department of Pharmacology All India Institute of Medical Sciences(AIIMS) Rishikesh India; ^2^ Department of Radiation Oncology All India Institute of Medical Sciences(AIIMS) Rishikesh India; ^3^ Department of Hemato‐Oncology All India Institute of Medical Sciences(AIIMS) Rishikesh India; ^4^ Department of Psychiatry All India Institute of Medical Sciences(AIIMS) Rishikesh India

**Keywords:** cancer chemotherapy, domains, FACT‐G, quality of life (QoL), tertiary care hospital

## Abstract

**Background:**

Cancer is the second most common cause of deaths worldwide. Likewise, in India, it is a major health problem, and disease burden is escalating every year. Cancer chemotherapy produces unfavorable effects on the well‐being of an individual. Since the past few years, quality of life (QoL) is considered as the main goal of cancer treatment in the survival of a patient.

**Aim:**

This current study aimed to assess the QoL and factors affecting it in adult cancer patients undergoing chemotherapy treatment.

**Methods and Results:**

An analytical, cross‐sectional study was conducted to achieve the objectives, employing the consecutive sampling method. A total of 120 adult (>19 years) patients were recruited from daycare chemotherapy unit of a tertiary care hospital. The data were collected using patient record form and Functional Assessment of Cancer Therapy‐General (FACT‐G), a quality of life (QoL) questionnaire. The overall mean score of quality of life (QoL) was 61.933 ± 5.85502. The domains of functional well‐being and emotional well‐being were most negatively affected after cancer chemotherapy. Education (illiteracy) and occupation (unemployment) were negatively associated with overall quality of life (QoL) of cancer patients on chemotherapy. Adverse drug reactions due to cancer chemotherapy negatively affect the quality of life (QoL) of cancer patients. Education (illiteracy) affects social well‐being domain of cancer patients. Working in the government/private sector has a positive impact on functional well‐being domain of quality of life (QoL).

**Conclusion:**

The study findings suggest an overall low quality of life (QoL) among adult cancer patients undergoing chemotherapy at our setup. It has been identified as a stressful therapy, also affecting both psychological and physical well‐being. Poor infrastructure, illiteracy, poverty, and lack of proper treatment facilities at most centres often lead to poor survival outcomes and hence focus has always been on achieving quantity of life rather than quality of life (QoL). This is further complicated due to nonavailability of validated tools in local vernacular, apathy of the treating physicians in the context of QoL aspects and social and cultural factors that are unique to this society. Psycho‐oncology needs to become an integral entity of comprehensive cancer care.

## 1 INTRODUCTION

Worldwide, deaths due to malignancy are the second common leading cause of mortality. Globally, about one in six deaths is due to malignancy. Roughly, 70% of fatality from cancers occur in low‐ and middle‐income countries.^1^ Cancer mortality in India has increased twofold from 1990 to 2016.^2^ Cancer in India has approximated 1.15 million new cancer patients in 2018 and is anticipated to almost double as a result of demographic changes alone by 2040.^3^

Classical end points in clinical therapeutic trials in oncology have been usually defined by total, recurrence‐free, or systemic disease–free survival. Primarily, these allow an adequate description of the biological course of the disease. In current oncological research, positive developments in the management of cancer patients as measured by such end points must actually be presumed to be only of a rather small magnitude. This signifies, on the one hand, that prospectively only by large‐scale multicentre trials and retrospectively only by meta‐analyses of commensurate trials can adequate numbers of patients be reached to allow a detection of treatment benefits of a pragmatic size. On the other hand, if treatments do not differ much with respect to survival, it is a reasonable step forward to stretch out the classical criteria of assessing treatment efficacy. It is undisputed that the disease and its treatment have a bearing on all aspects of a cancer patient's life. Although physicians had previously recorded the occurrence of toxic reactions possibly induced by cancer treatment, it was not until the 1940s that with their pioneering work, Karnofsky et al (1948)^4^ made a first attempt to quantify the performance status of patients with advanced cancer. In the ensuing decades, there has been increasing recognition of the need to achieve a more comprehensive evaluation of treatment efficacy over and above the objective facets of achieving optimal survival, maximal tumor response, and minimal toxicity (Maguire & Selby, 1989).^5^ In attempting to reach this target, additional end points in cancer clinical trials were initiated that embrace the subjective response of the patients to their illness and its treatment. The sum of aspects of the patients' subjective well‐being is most often called “quality of life”(QoL) (eg, Tchekmedyian & Cella, 1990).^6^

Although each individual usually has an instinctive comprehension of what quality of life implies for him or her, no general and unique definition of QoL is deemed as conceivable or even sensible. Cella and Tulsky (in Tchekmedyian & Cella, 1990)^6^ honestly confess that “QoL cannot be validly measured because it means so many different things to so many different people.” This is particularly valid for the remarkable life situation of persons who have faced the often life‐threatening detection of cancer.

Instead of trying to garnish an analytical definition, QoL is initiated into clinical research by means of a so‐called operational construct, recognizing that an individual's life and its matching quality cannot be quantified in an objective way. Instead, and rather pragmatically, a patient's QoL is quantified using measurements obtained from a set of sensibly defined, quantifiable dimensions. The major points of settlement among QoL researchers on this construct can be epitomized by the statements that QoL is:

1. Multidimensional: encompassing important elements of a patient's emotional, social, and physical well‐being;
2. Subjective: depending on primarily on the patient's own judgments; and
3. Nonstatic: and subject to transitions over a patient's lifetime.

Point 1 leads to the requirement that QoL has to be dissected into its major aspects, each of which can be sufficiently concretized for an evaluation in patients. A suitable measuring instrument should account for the multidimensionality of QoL by satisfactorily embracing all the major dimensions. The second point of the QoL construct seems rather obvious, but it has taken some time to become established that, whenever possible, the individual patient is the principal authority to be asked about his/her QoL. Physician's assessment of the patients' QoL, which was widely resorted to when QoL methodology was introduced, has proved to be less trustworthy when used exclusively (Slevin et al, 1988; Regan et al, 1991).^7^, ^8^ It is irrefutable that a detailed interview is the most suited approach to inclusively evaluate an individual's well‐being. However, the most workable form of a measuring instrument in the background of multicenter trials is the self‐administered questionnaire. A good questionnaire is distinguished by having in it certain so‐called “psychometric” standards like validity (measuring what is intended to be measured), reliability (measuring with sufficient precision), and sensitivity (ability to detect changes). The last is important especially in the light of point 3 of the QoL construct. A person's QoL is subject to transitions over time, evincing, for example, the patient's competency to cope with the disease or the experiences with varied treatment modalities. Therefore, a requisite evaluation of QoL is imperative at more than two points in time to be able to evaluate both short‐ and long‐term effects of treatments. Because of the need to setup and use valid and reliable measuring instruments, the endorsement of any preexisting validated questionnaires should be selected over the development of new ad hoc questionnaires. If one feels that important specific aspects are absent in a specific questionnaire, it is in most cases possible to append ancillary components to the existing measuring instrument without altering its original structure.^9^

The term quality of life (QoL) is used to assess the general well‐being of a person and society. According to the World Health Organization (WHO), quality of life (QoL) has been defined as an individual perception of life, values, objectives, standards, and interests in the framework of culture. It is the subjective evaluation of life as a whole or the patient's appraisal and satisfaction with their current level of functioning compared with what they perceive to be possible or ideal. Quality of life (QoL) is a multidimensional construct capturing the subjective well‐being (both positive and negative aspects) of patients in physical, emotional, functional, and social domains (or dimensions). Several illness‐related factors exist that can affect QoL. The extent of distress symptoms experienced by a person has been related to QoL in many people with cancer. QoL is more and more being utilized as essential outcome measures in studies to decide the efficacy of treatment.^10^, ^11^

All domains of an individual's QoL can be affected by malignancy. The deterioration in the QoL kicks off following diagnosis of the malignancy and lingers due to the vigorous nature of the treatment. Cancer patients receive chemotherapy to fight against the affliction. Out of 65% of the cancer population, chemotherapy is being used as the first line of management in 25% of patients.^12^ Anticancer drugs target the rapidly dividing abnormal cells, thereby helping to combat cancer and promoting the survival of patients. Despite chemotherapy having a therapeutic effect, it is associated with the development of severe unfavorable drug reactions which can have inimical effects on the QoL of an individual. Moreover, anticancer therapy requires an extended duration of administration to obtain the required effect. Frequent hospitalizations put an undue burden on cancer patients. Thus, anticancer therapy engenders a colossal personal, mental, and emotional anguish among cancer individuals, affecting their overall QoL. Findings of QoL research provide information about the effect of disease and its treatment on functioning and well‐being, recognizing common problems and designing appropriate approaches to resolve these issues. QoL research findings help the physicians to appreciate the effect of chemotherapy on patients' well‐being and predicting the therapy response.^13^

QoL has been a less explored dimension among cancer patients in India. Available work on posttreatment QoL has mostly been documented in Western literature and less in Indian literature.^14^ A study by Kannan et al (2011) in South India concluded that 80% of cancer patients have average and below average QoL.^15^ Sundarem et al (2016) conducted a study to assess QoL in relation to treatment, and he concluded that cancer patients undergoing chemotherapy had poor QoL.^16^ In a study by Elsaie et al (2012), it was found that there was a significant decrease in functional well‐being of colorectal cancer patients after chemotherapy.^17^ D'souza et al (2013) conducted a study on head and neck cancer patients and concluded that physical, psychological, and functional domains are affected.^18^ In a cross‐sectional study conducted by Jacob et al (2019) in India, lower‐income status had a positive association with decreased physical, social, and psychological well‐being.^19^ A cross‐sectional study on breast cancer patients in India showed overall QoL to be good except for sexual function.^20^ Most of the QoL studies have been conducted only in the southern and eastern region of India. There are very few notable studies about QoL research in North India. A study by Sharma et al(2017) in Delhi concluded that there is association between clinical, sociodemographic characteristics, and QoL of breast cancer patients.^21^ Another study by Barwal et al (2016) in North India concluded that there is a decline in the global QoL and social functioning in lung cancer patients following treatment.^22^

Uttarakhand is now recognized as India's “cancer bowl” with rising cancer burden leading to additional noncommunicable disease load. This is one of the states which has high crude cancer incidence rates (91.0 per 1,00000) in 2016 according to India State‐Level Disease Burden Initiative.^23^ There are various cultural, ethnic diversities existing among different regions of India. Uttarakhand and its associated hilly areas have a varied diverse heterogeneous population. A study by Mason et al (2019) showed that 39% of cancer patients had psychological distress, and prevalence was higher in female patients and old‐age patients.^24^ Another study by Pruthi et al (2018) showed that emotional well‐being was affected negatively after chemotherapy in surgically resected gastric cancer patients. QoL is the least explored dimension in this part of the country which has a high disease burden.^25^ Hence, this study was conducted to explore the QoL of adult cancer patients on chemotherapy in the hilly Himalayan region of the northern part of India.

## 2 METHODS

### 2.1 Purpose

To determine the overall QoL and factors affecting it in adult cancer patients undergoing cancer chemotherapy in Uttarakhand region.

### 2.2 Research design

This was a cross‐sectional, analytical study.

### 2.3 Research questions

What was the general level of QoL in physical, social, emotional, and functional domains in adult cancer patients?

What were the factors that affect QoL in adult cancer patients?

### 2.4 Study setting and participants

Participants were recruited using consecutive sampling strategy from January 2019 to June 2019. The study was conducted in the Oncology Daycare Centre attached to the Department of Radiotherapy & Hemato‐Oncology, AIIMS, Rishikesh. Patients were recruited after explaining to them the purpose, benefits, and risks of the study. Participants were selected based on the following inclusion criteria: (a) patients who were 19 years of age and older, (b) patients who received cancer chemotherapy (two or more cycles), and (c) patients able to speak and understand Hindi or English language. Patients afflicted with diabetes, cardiac diseases, and mental illness were excluded from the study.

**FIGURE 1:**
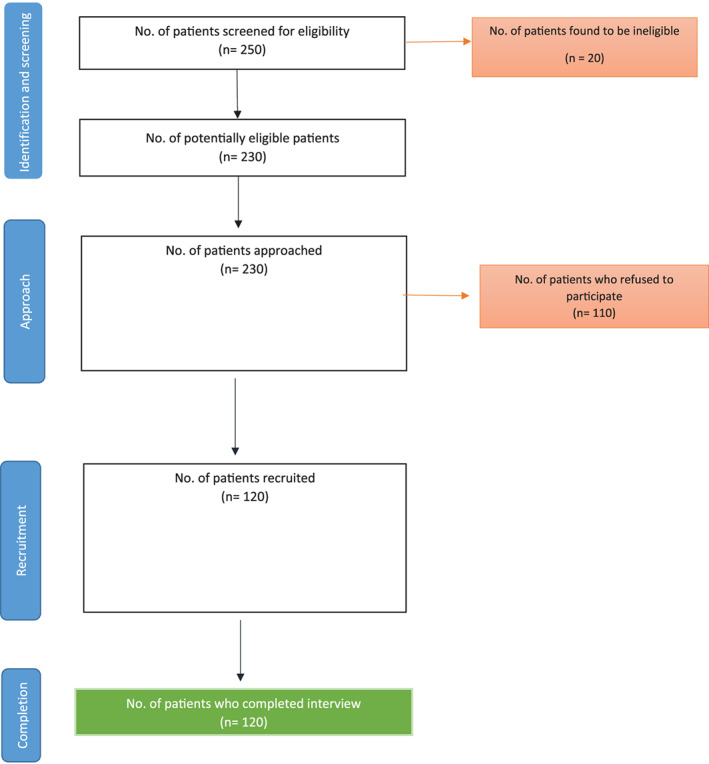
Patient recruitment log

We screened 250 patients for the eligibility criteria, and of them, 230 patients were identified as eligible (Figure 1). Out of 230 patients, 110 refused to be part of the study due to a lack of interest. Finally, 120 patients were included, and they completed the interview and questionnaire.

### 2.5 Ethical considerations

This study was conducted after obtaining due approval from Institutional Ethics Committee (IEC)(AIIMS/IEC/18/161 dated 4.1.2018).

### 2.6 Measures

#### 2.6.1 Data collection tools

Data were collected from the study participants using a specially designed Patient Record Form prepared by the investigators. This customized form included questions about sociodemographic features, clinical characteristics and treatment details of the patients, and Functional Assessment of Cancer Therapy‐General (FACT‐G), quality of life (QoL) questionnaire, which characterized cancer patients' QoL.

#### 2.6.2 Functional assessment of cancer therapy‐general (FACT‐G)

This questionnaire was developed by an organization, Functional Assessment of Chronic Illness Therapy (FACIT) measurement system to assess the QoL of all types of cancer patients.^26^ This questionnaire consists of 27 questions measured on a five‐point Likert Scale. The questionnaire consists of four QoL domains (dimensions): physical, social/family, emotional, and functional well‐being. The higher the score, the better the QoL.

The reliability and validity of FACIT QoL tool were ascertained in gall bladder patients in India. The Cronbach's α for the total scale was found to be 0.85 indicating high internal consistency, and for the subscales, it ranged from 0.566 to 0.782.^27^

The original FACT‐G tool is in English, and a translated version in Hindi is also available. The Hindi translation of the questionnaire has been validated in Indian cancer patients by Singh.^28^ Prior permission was obtained from FACIT organization, and the validated Hindi translated tool was administered to the cancer patients.

### 2.7 Data analysis

All data were analyzed with the help of Statistical Package for the Social Sciences (SPSS) version 20.0. To compare the FACT‐G scores with those from US‐based studies and Indian cancer patients, we calculated Cohen's *D* effect sizes to compare the means from the two samples, and effect size 0.8 and over were considered to be large.^29^ Descriptive statistics, frequencies, percentages, and means were used to represent the data in connection with the sociodemographic profile, clinical characteristics of patients, and FACT‐G QoL scores. One‐way analysis of variance (comparison of means of three or more groups) was used to compare the mean QoL scores for several demographic and cancer treatment–related variables. *P* < .05 was considered to be statistically significant.

## 3 RESULTS

### 3.1 Demographic and disease characteristics

A total of 120 patients participated in the study and completed the questionnaire. Of the 120 patients, 65 (54.2%) were females and 55 (45.8%) were males. The mean age of the study subjects was 49.68 years. Sixty (50%) patients had a per capita income lower than Rs. 20000. Gastrointestinal cancers were most common among the patients accounting for 31.7% followed by breast cancer (15.8%). Sixty two (51.7%) patients had two adverse drug reactions (ADRs) and 45 (37.5%) had one ADR. Forty seven (39.2%) patients presented with stage 2 cancer, while 43 (35.8%) patients had stage 3 cancer (Table 1).

**TABLE 1:** Distribution of demographic, disease, and treatment variables (n = 120)

Variable	Grouping	Frequency(n)	Percentage(%)
Age	19‐40	30	25
	40‐50	29	24.2
	50‐60	25	20.8
	>60	36	30
Sex	Female	65	54.2
	Male	55	45.8
Income status	Rs. <20 000	60	50
	Rs. 20 001‐50 000	58	48.3
	Rs. >50 000	2	1.7
Education	Illiterate	28	23.3
	Primary/secondary	61	50.8
	Collegiate	31	25.8
Occupation	Business/agriculture	29	24.2
	House wife	37	30.8
	Government/private employees	52	43.33
	Unemployed	2	1.67
Cancer type	Haematological	18	15
	Breast	19	15.8
	Gastrointestinal	38	31.7
	Lung	14	11.7
	Lymphoma	14	11.7
	Cervix	6	5
	Others	11	9.2
Type of treatment	Chemotherapy	81	67.5
	Chemo radiotherapy	39	32.5
No. of ADRs	1	45	37.5
	2	62	51.7
	3	12	10
	4	1	0.8
Stage of the disease	1	17	14.2
	2	47	39.2
	3	43	35.8
	4	13	10.8

### 3.2 Findings on QoL outcomes

The total FACT‐G QoL mean score was 61.933 ± 5.85502. An evaluation of subscale mean scores shows that physical well‐being (min: 0‐max: 28) was 17.39 ± 2.692, social well‐being (min: 0‐max: 28) was 15.95 ± 3.493, emotional well‐being (min:0‐max: 24) was 14.50 ± 2.158, and functional well‐being (min:0 ‐ max:28) was 13.95 ± 3.295; the subscale which was most negatively affected was functional well‐being followed by emotional well‐being (Table 2).

**TABLE 2:** Quality‐of‐life scores in the four domains of the Functional Assessment of Cancer Therapy‐General tool

Domains	n	Minimum	Maximum	Mean	SD
Physical well‐being	120	9	25	17.39	2.692
Social/family well‐being	120	3	26	15.95	3.493
Emotional well‐being	120	8	22	14.50	2.158
Functional well‐being	120	4	21	13.95	3.295
Total QOL	120	46	81	61.933	5.85502

Compared to US patients, the reported general well‐being scores were lower in all subscales, especially for social well‐being (effect size[ES] = 1.2; CI: 1.3‐1) and emotional well‐being (ES = 1; CI: 1.1‐0.8), indicating worse QoL than the US sample (Table 3).

**TABLE 3:** Comparison of QoL outcomes with US cancer sample

Instrument	Uttarakhand patients (N = 120)	US cancer sample (N = 2236)^a^	Cohen's *D*
	Mean (SD)	Mean (SD)	Mean (95% CI)
FACT‐G total score	61.9(5.8)	80.9(17.0)	−1.1(−1.3, −0.9)
FACT‐G PWB subscale	17.4(2.7)	21.3(6.0)	−0.7(−0.8, −0.5)
FACT‐G SWB subscale	16.0(3.5)	22.1(5.3)	−1.2(−1.3, −1)
FACT‐G EWB subscale	14.5(2.1)	18.7(4.5)	−1(−1.1, −0.8)
FACT‐G FWB subscale	13.9(3.2)	18.9(6.8)	−0.7(−0.9, −0.6)

Abbreviations: EWB, emotional well‐being; FWB, functional well‐being; PWB, physical well‐being; SWB, social well‐being.

^a^ FACT‐G is referenced from Brucker PS, Yost K, Cashy J, Webster K, Cella D. General population and cancer patient norms for the Functional Assessment of Cancer Therapy‐General (FACT‐G). Evaluation and the health professions. 2005 June; 28 (2):192‐211.

Compared to Hyderabad patients, the reported overall well‐being scores was slightly lesser. The emotional well‐being (effect size [ES] = 1.7; CI: 1.9‐14) and functional well‐being (effect size [ES] = 1.3; CI: 1.0‐1.5) scores were much lesser in Uttarakhand patients compared to Hyderabad patients (Table 4).

**TABLE 4:** Comparison of QoL outcomes with Hyderabad cancer sample

Instrument	Uttarakhand patients (N = 120)	Hyderabad patients (N = 210)^19^	Cohen's *D*
	Mean (SD)	Mean (SD)	Mean(95% CI)
FACT‐G total score	61.9(5.8)	62.4(10)	−0.05(−0.2, 0.6)
FACT‐G PWB subscale	17.4(2.7)	17.0(4.5)	0.1(−0.1, 0.3)
FACT‐G SWB subscale	16.0(3.5)	16.2(3.3)	−0.02(−0.2, 0.1)
FACT‐G EWB subscale	14.5(2.1)	20.0(3.7)	−1.7(−1.9, −1.4)
FACT‐G FWB subscale	13.9(3.2)	9.2(3.8)	1.3(1.0, 1.5)

Abbreviations: EWB, emotional well‐being; FWB, functional well‐being; PWB, physical well‐being; SWB, social well‐being.

### 3.3 Factors affecting the quality of life

A comparison of FACT‐G overall QoL score and its subscale mean scores for some variables about demographic features and cancer treatment was done. The overall FACT‐G mean QoL score for illiterate patients was significantly low (*P* = .009) and also for those who were engaged in agriculture/business (*P* = .04). No significant differences were found when FACT‐G overall mean QoL scores were compared in terms of age, income status, type of cancer, number of ADRs, and stage of the disease (Table 5).

**TABLE 5:** Comparison of means (with relevant F values) pertaining to overall and subscale scores QoL FACT‐G scores

Variable Age(years)	FACT‐G Quality of Life scale					
	Group	Physical well‐being	Social well‐being	Emotional well‐being	Functional well‐being	Total
19‐40	1	17.20	17.10	14.90	14.33	63.53
40‐50	2	17.17	15.93	14.83	13.79	61.72
50‐60	3	17.88	16.32	13.92	14.12	62.24
>60	4	17.39	15.22	14.31	13.64	60.55
‐		F= 0.383	F= 1.86	F=1.275	F= 0.281	F= 1.463
‐		p= 0.766	p= 0.139	p= 0.286	p= 0.839	p= 0.228
**Sex**						
Male	1	17.68	16.03	14.40	13.51	61.62
Female	2	17.05	16.16	14.62	14.47	62.30
		p= 0.208	p= 0.827	p= 0.583	p= 0.110	p= 0.520
**Education**						
Illiterate	1	16.64	15.00	14.71	13.04	59.39
Primary/Secondary	2	17.54	16.02	14.26	14.23	62.04
Collegiate	3	17.77	17.23	14.77	14.23	64.00
‐		F= 1.502	F= 3.516	F= 0.755	F= 1.416	F= 4.87
‐		p= 0.227	p= 0.033^*^ (D = 1 from 3)	p=0.472	p= 0.247	p= 0.009^**^ (D = 1 from 2,3)
**Income status**						
Rs. <20000	1	17.33	15.73	14.43	13.65	61.15
Rs. 20001 – 50000	2	17.45	16.33	14.64	14.26	62.67
Rs. >50000	3	17.50	20.00	12.50	14.00	64.00
‐		F= 0.028	F= 1.931	F= 1.006	F= 0.499	F= 1.126
‐		p= 0.972	p= 0.15	p= 0.369	p= 0.608	p= 0.328
**Occupation**						
Business/agriculture	1	17.93	15.48	14.31	12.34	60.06
Housewife	2	16.97	15.92	14.49	14.38	61.75
Government/private employees	3	17.44	16.73	14.48	14.67	63.32
Unemployed	4	16.00	11.50	18.00	10.50	56.00
‐		F= 0.868	F= 2.380	F= 1.871	F= 4.420	F= 2.77
‐		p= 0.460	p= 0.07	p= 0.138	p= 0.006^*^ (D = 1 from 2,3)	p= 0.04^*^ (D = 1 from 3)
**Cancer type**						
Haematological	1	17.39	16.83	15.00	13.78	63.00
Breast	2	16.68	16.37	14.89	14.16	62.10
Gastrointestinal	3	17.68	15.42	14.68	13.89	61.68
Lung	4	17.00	15.29	13.50	14.21	60.00
Lymphoma	5	17.79	18.07	14.14	13.71	60.72
Cervix	6	17.67	15.67	14.67	14.33	63.71
Others	7	17.45	15.45	14.00	13.82	62.33
‐		F= 0.392	F= 1.537	F= 0.982	F= 0.063	F= 0.65
‐		p= 0.883	p= 0.172	p= 0.441	p= 0.999	p= 0.68
**No.of.ADRs**						
1	1	17.51	16.16	14.38	13.47	61.51
2	2	17.44	16.39	14.35	14.45	62.62
3	3	17.42	14.58	16.25	12.67	60.91
4	4	9.00	13.00	8.00	20.00	50.00
‐		F= 3.47	F= 1.307	F= 6.620	F= 2.637	F= 1.91
‐		p= 0.018^*^ (D = 4 from 1,2,3)	p= 0.275	p= 0.000^***^ (D = 4 from 1,2,3)	p= 0.053	p= 0.130
Stage of the disease						
I	1	17.76	17.41	13.53	14.41	63.11
II	2	17.34	16.17	14.85	13.74	62.10
III	3	16.69	16.07	14.49	14.09	62.16
IV	4	17.39	14.15	14.54	13.62	59.00
‐		F= 0.429	F= 2.49	F= 1.586	F= 0.239	F= 1.368
‐		p= 0.733	p= 0.06	p= 0.196	p= 0.869	p= 0.256

^*^ p<0.05,

^**^ p<0.01,

^***^ p<0.001, ‘D’ indicates the group(s) that significantly differ.

The physical well‐being score of patients who have more number of ADRs was found to be significantly low (*P* = .018) (Table 5). The social well‐being mean scores of patients who never went to school (illiterate) were found to be significantly low (*P* = .033) (Table 5). The emotional well‐being mean scores of the patients who have more ADRs were significantly low (*P* = .000) (Table 5). The functional well‐being subscale mean scores for patients who were engaged in agriculture/business were significantly low (*P* = .04) (Table 5).

## 4 DISCUSSION

The present study reveals a mean score of QoL as 61.93 which represents low QoL among adult patients undergoing cancer chemotherapy. Upon comparison with US patient sample, patients in our sample reported lower functional, physical, emotional, and social well‐being. This disparity in wellness scores may be explained by the availability of well‐resourced allied health facilities in high‐income countries and the relative lack of these facilities in our study setting and Indian public health context in India. The mean QoL score on our study is lesser when compared to other studies conducted in India.^19^, ^30^ The low QoL is attributed to the patient population who presented to the hospital with advanced stage of the disease.^31^ Forty seven patients were diagnosed with stage 2 of the disease and 56 patients with stage 3 and 4 of the disease. Eighty two percentage of population in Uttarakhand belongs to Hindu religion, and they have spiritual concerns which are linked to poor psychosocial health and poorer QoL.^32^ The accessability to healthcare system in Uttarkhand is also a reason for low QoL because of the shortage of physicians in remote and farflung hilly areas.^33^

Though females had lesser mean QoL scores than males, the gender did not affect the overall QoL of patients in our study (*P* > .05). Two other QoL studies in cancer patients have reported similar results.^34^, ^35^ On the other hand, in two other studies, female patients had lower physical, social, psychological life qualities.^36^, ^37^

Within all the domains, functional well‐being was most commonly affected in our study subjects, the score being 13.95. The functional well‐being scale involves those items which reflect the capability to work and function independently. Out of the four domains, the respondents opined that the functional domain had a great impact on their QoL. These findings are congruent with a previous study which expressed autonomy as one of the crucial indicators of QoL among people with long‐standing osteoarthritis, rheumatoid arthritis, and diabetes.^38^ Chemotherapy can reduce the functional capacity of cancer patients and also increase the level of fatigue in cancer survivors.^39^

The second most affected QoL domain in our study was emotional well‐being with a mean score of 14.50. Our findings are consistent with a study conducted on Indian cancer patients.^19^ Fear of cancer recurrence and related depressive symptoms plays a crucial role in dealing with the disease and the recovery process. Our findings are in contrast to the popular belief that Indian patients are well‐protected with a tight family network. The identification of cancer, chemotherapy cycles, and cancer relapses may all be grounds for considerable stress and upheavals necessitating psychosocial adaptations, both for family and patients.

Cancer diagnosis alters the family functioning and imposes a financial strain on the family which might make the patient perceive a loss of family. Family members experience psychological stress which in turn causes problems in their job, including absence, a decrease in their productivity, threat of dismissal, and financial issues.^40^

Economic distress negatively affects the emotional well‐being of cancer patients. Likewise, fear and stigma associated with cancer also affect the emotional well‐being of the patient. The stigma of having cancer and the fear of discrimination and the denial of fundamental welfare rights lead to social exclusion.^41^ Malignancy‐related myths and stigma have effects on diagnosis and treatment. Posttreatment effects such as loss of hair and physical deformities results in undesirability, unacceptability, and proclivity to stigma. Fright of death in cancer makes the patient vulnerable toward social separation.

In our study, illiterate patients had significant negative QoL when compared to their educated counterparts. Several studies (including one from India) have reported an association between educational level and QoL.^42^, ^43^ Education is one of the important factors that help in promoting QoL. More trained patients need less time and energy from doctors than illiterate patients in terms of diagnosis and follow‐up care.^44^ Higher education provides knowledge, better understanding, and awareness creation, which ultimately improve the overall QoL.^45^ Moreover, importance should be given to health literacy which increases the health awareness of the patients. Health literacy is linked to literacy and entails people's knowledge, motivation, and competences to access, understand, appraise, and apply health information in order to make judgments and take decisions in everyday life concerning healthcare, disease prevention, and health promotion to maintain or improve quality of life during the life course.^46^ Patients should have the ability to apply reading and numeracy skills in a healthcare setting. The level of health awareness is low in Indian population due to lower educational status, poor functional literacy, and less priority for health.^47^

From this study, it is evident that the number of ADRs impacts the physical well‐being and emotional well‐being of cancer patients. Most of the patients experienced nausea, pain, and lack of energy due to chemotherapy which might be the reason for negative physical well‐being of the patients. These findings are also in line with previous studies.^48^, ^49^ On account of the ADRs, most of the patients feel sad, with a sense of hopelessness in fighting against cancer, which ultimately affects the emotional well‐being of a patient.

A significant positive association was seen between the group of government/private employees and overall QoL. Similar findings have been reported in studies conducted by Safaee et al.^50^ Studies also show a contradictory association between employment status and QoL.^51^ Employment may provide financial means to control the disease, but it can worsen the QoL owing to frequent hospital visits and workload. While unemployed patients may face financial difficulties, they may attend hospital visits in a more comfortable way than those who are employed. Besides, friends and colleagues on the worksite can also play a crucial role in improving the QoL.

The long‐lasting ADRs of cancer chemotherapy and the treatment of cancer‐related symptoms are important areas for physicians and cancer patients to ponder upon when attempting to recognize and enhance the QoL effects.^52^

Educational programs for doctors and other healthcare workers who are dealing with cancer patients can foster better interaction between patients and healthcare workers. Hence, they can willingly tap active cooperation; patients and their families can better deal with treatment‐associated ADRs thereby enhancing better social and functional well‐being.

Most of the cancer patients avail spiritual help as their crucial coping mechanism. Some studies have documented that the greater use of positive religious coping improves the overall QoL of cancer patients.^53^

In North India, very few studies have been undertaken to focus on QoL concerns of cancer subjects. Poor infrastructure, illiteracy, poverty, and lack of proper treatment facilities at most centres often lead to poor survival outcomes, and hence focus has always been on achieving quantity of life rather than quality of life (QoL). This is further complicated due to nonavailability of validated tools in local vernacular, apathy of the treating physicians in the context of QoL aspects and social and cultural factors that are unique to this society. Psycho‐oncology needs to become an integral entity of comprehensive cancer care.^54^, ^55^ Therefore, the results of our study are intended to inform and motivate healthcare workers and investigators for undertaking further analysis and outlining effective patient care plans to enhance QoL.

## 5 LIMITATIONS

The modest sample size is a limitation of this study. The modest sample size might have caused some uncertainty during subgroup analysis. Longitudinal studies are underway to provide a clearer picture of QoL in patients undergoing anticancer therapy in North India.

## 6 CONCLUSIONS

The findings of the present study indicate low QoL among cancer patients on anticancer therapy. Functional well‐being was most affected in cancer patients followed by emotional well‐being among cancer patients on anticancer therapy. From our study, it was found that the overall QoL of patients was influenced by education and occupational status of the patient. Unemployed and illiterate patients have worser QoL than employed and educated patients. Chemotherapy‐induced ADRs affect the physical well‐being as well as the emotional well‐being of patients. It is imperative to initiate assuasive programs for patients on cancer chemotherapy in order to mollify their physical and emotional sufferings and consequently improving the QoL.

## CONFLICT OF INTEREST

The authors declare no potential conflict of interest.

## AUTHORS' CONTRIBUTIONS

All authors had full access to the data in the study and take responsibility for the integrity of the data and the accuracy of the data analysis. *Conceptualization*, R.S.K., V.S.R., B.D.; *Data Curation*, R.S.K., B.D.; *Formal Analysis*, R.S.K., B.D.; *Methodology*, R.S.K., R.K.P., U.N., V.S.R., B.D.; *Project Administration*, R.S.K., R.K.P., U.N., B.D.; *Software*, R.S.K.; *Writing‐Original Draft*, R.S.K.; *Resources*, R.K.P., B.D.; *Supervision*, R.K.P., U.N., B.D.; *Validation*, V.S.R.; *Writing‐Review & Editing*, V.S.R., B.D.; *Investigation*, B.D.

## ETHICAL STATEMENT

This study was conducted after obtaining due approval from Institutional Ethics Committee(IEC) (Sanction Letter No. AIIMS/IEC/18/161 dated 4.1.2018). Written informed consent was obtained from all study participants.

## Data Availability

The data that support the findings of this study are available on reasonable request from the corresponding author. The data are not publicly available due to privacy or ethical restrictions.
